# Ultra‐Low Threshold Resonance Switching by Terahertz Field Enhancement‐Induced Nanobridge

**DOI:** 10.1002/advs.202405225

**Published:** 2024-11-04

**Authors:** Sang‐Hun Lee, Moohyuk Kim, Yeeun Roh, Myung‐Ki Kim, Minah Seo

**Affiliations:** ^1^ Department of Optical Engineering Kumoh National Institute of Technology 350‐27, Gumidae‐ro Gumi Gyeongbuk 39253 Republic of Korea; ^2^ KU‐KIST Graduate School of Converging Science and Technology Korea University Anam‐ro 145, Seongbuk‐gu Seoul 02841 Republic of Korea; ^3^ Sensor System Research Center Korea Institute of Science and Technology Seoul 02792 Republic of Korea

**Keywords:** nanotip, nonlinear effect, quantum photonics, terahertz spectroscopy

## Abstract

Ongoing efforts spanning decades aim to enhance the efficiency of optical devices, highlighting the need for a pioneering approach in the development of next‐generation components over a broad range of electromagnetic wave spectra. The nonlinear transport of photoexcited carriers in semiconductors at low photon energies is crucial to advancements in semiconductor technology, communication, sensing, and various other fields. In this study, ultra‐low threshold resonance mode switching by strong nonlinear carrier transport beyond the semi‐classical Boltzmann transport regime using terahertz (THz) electromagnetic waves are demonstrated, whose energy is thousands of times smaller than the bandgap. This is achieved by employing elaborately fabricated 3D tip structures at the nanoscale, and nonlinear effects are directly observed with the THz resonance mode switching. The nanotip structure intensively localizes the THz field and amplifies it by more than ten thousand times, leading to the first observation of carrier multiplication phenomena in these low‐intensity THz fields. This experimental findings, confirmed by concrete calculations, shed light on the newly discovered nonlinear behavior of THz fields and their strong interactions with nanoscale structures, with potential implications and insights for advanced THz technologies beyond the quantum regime.

## Graphical abstract

The induced current channel, generated under colossal terahertz field enhancement, creates a nanobridge leading to a frequency‐doubled resonance spectrum. This represents the first observation of resonance changes of the metamaterial by nonlinear carrier transport of Si using the most widely used terahertz system, distinct from previous reports that utilized extremely intense experimental environments.

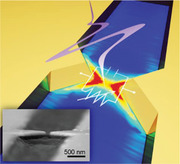

## 1 Introduction

Terahertz (THz) techniques have been widely used not only for industrial applications, such as nondestructive tests and security imaging, but also for the scientific analysis of macroscopic molecular vibrations,^[^ ^1^, ^2^ ^]^ excitons,^[^ ^3^ ^]^ phonons,^[^ ^4^, ^5^ ^]^ and superconductivity.^[^ ^6^, ^7^ ^]^ Unlike frequency ranges above the infrared region, THz waves have photon energies thousands of times smaller than the bandgaps of semiconductor materials that are commonly used to fabricate photodiodes. In addition, because the energy levels of THz waves are lower than the thermal energy at room temperature, direct measurement using bolometric and pyroelectric methods still shows insufficient sensitivity and responsivity, as well as large noise without cryogenic conditions,^[^ ^8^, ^9^ ^]^ even with plasmonic structures or low‐dimensional materials.^[^ ^10^, ^11^, ^12^, ^13^, ^14^, ^15^, ^16^ ^]^ Thus, THz systems with direct measurements based on solid‐state electronics are limited to the imaging applications coupled with intense sources in a restricted frequency range. A novel approach for broadband devices based on photonic techniques is required for advanced practical applications using the spectroscopic advantages of THz waves.

Under the most widely used broadband THz systems with a photoconductive antenna, which shows the emission in the level of nW,^[^ ^8^ ^]^ the interaction between THz waves and photoexcited carriers in semiconductor materials typically follows a linear response, where the behavior of carriers is directly proportional to the applied weak THz field. In this regime, the carrier dynamics can be described using linear models such as Drude theory or the semi‐classical Boltzmann transport equation.^[^ ^17^, ^18^ ^]^ Nonlinear carrier dynamics for sensitive material response to THz waves require more significant field strengths.^[^ ^19^, ^20^, ^21^ ^]^ Such cases have typically been introduced using the femtosecond laser‐based amplifier systems with the tilted‐pulse‐front scheme using a LiNbO_3_ wedge, which can generate an intense THz field up to the MV/cm range.^[^ ^22^ ^]^ These intense field can result in various physical phenomena, including nonlinear carrier transport phenomena (e.g., Zener tunneling,^[^ ^23^ ^]^ impact ionization,^[^ ^23^, ^24^ ^]^ and intervalley scattering^[^ ^19^, ^20^, ^24^ ^]^) leading to various nonlinear optical effects.^[^ ^25^ ^]^ However, thus far, it has been strictly proven that a low‐intensity THz field below the threshold alone does not induce such strong nonlinear carrier effects without additional field‐enhancing tools.

From this perspective, nanoscale patterns, such as gap arrays, help modify the surface properties of materials by manipulating the redistribution of the local electromagnetic field.^[^ ^26^, ^27^, ^28^, ^29^, ^30^, ^31^ ^]^ These patterns can lead to localized and collectively excited carriers and strong interaction with incident light, causing substantial field enhancement as well, under an extremely shrunken regime.^[^ ^25^, ^32^, ^33^, ^34^, ^35^ ^]^ Given the strong interaction between excited carriers and incident fields, nonlinear effects that foster carrier transport pathways arise.^[^ ^21^, ^25^, ^32^, ^33^, ^34^, ^36^ ^]^ Nevertheless, a strong THz field, which is based on a rather complicated and bulky system with a regenerative amplifier, is still required to surmount the threshold and generate nonlinear carrier transport inside the field hotspot of metamaterials, making broad application difficult.

In this study, we demonstrate the switching of the THz resonance mode through strong nonlinear phenomena beyond the Boltzmann transport equation with an exceptionally low threshold of incidence THz fields. In the elaborately defined nanoscale bowtie tip for inducing field‐dependent topological bridging at the center of the nanoslot, the THz electric field was intensively localized and amplified by over ten thousand times. This phenomenon, caused by the squeezed mode volume, results in carrier multiplication in the Si substrate, which is triggered by the incident THz field at just a few tens of V/cm. Through carrier multiplication, the presence of an instantaneous current across the gap between nanotips suppresses the fundamental resonance and causes a slight blue shift at the resonance frequency, operating similarly to pinch harmonics. Nanoscale pattern‐enabled effects under a low‐intensity THz field can provide unique platforms for tailoring and controlling the transport of photoexcited carriers at the nanoscale from a completely new perspective. This observation has implications in various fields, including optoelectronics, photovoltaics, and quantum information processing, where the precise control of carrier dynamics is desired.

## 2 Result

### 2.1 Nonlinear Carrier Transport Using Nanotips

**Figure 1:**
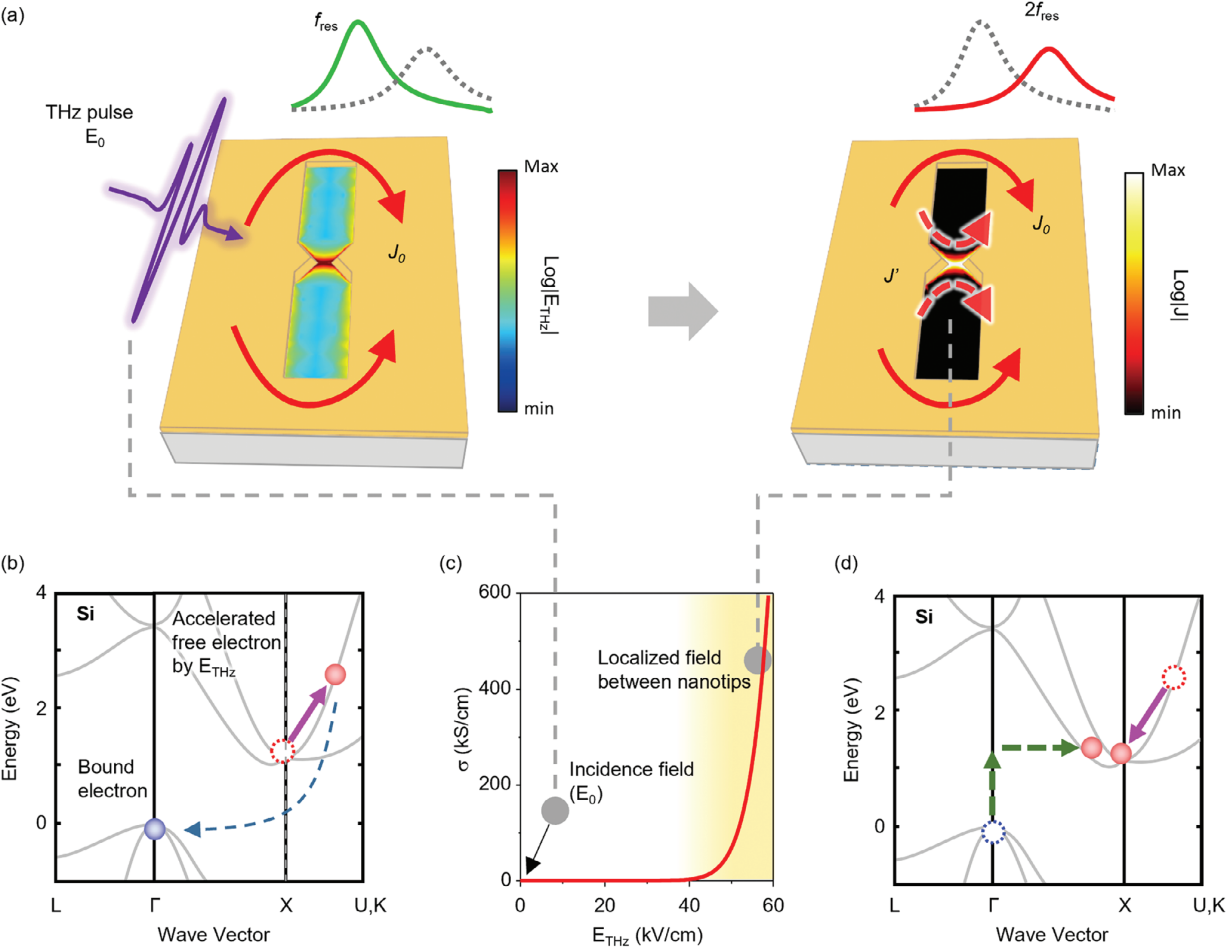
a) The incident THz field, with an intensity of just a few tens of V/cm, causes resonance in which the surface current is induced over the metal film. With assistance of a 3D nanotip structure, the induced current channel can be generated through an tiny gap, and the resonance spectrum is frequency‐doubled. b) Carrier transitions are represented in the Si band structure with free electrons accelerated by the incident THz field. c) The calculated induced conductivity change induced by the incident electric field. Gray dots match the resonant cases for the conditions below and above the threshold described in (a). d) Carrier transitions in Si band structure, representing the process of phonon‐assisted interband transition *Г*‐to‐*X* valley (green) and intraband electron relaxation through carrier multiplication (violet).

The 3D nanotip structure was applied to realize an extremely squeezed mode volume accompanied by a THz resonator, which has a fundamental resonance at 0.6 THz, *f_res_*, determined by the severely asymmetric geometry of the metallic slot (1 µm in width and 100 µm in length), applied on a Si substrate (**Figure** **1**a). Once the THz resonator induces a collective oscillation of the incident THz electromagnetic wave with polarization across the resonance structure, strong resonance behavior can occur. In this scheme, the surface current *J_0_* is induced over the metal film, rounding the corners of the resonators (Section S1, Supporting Information). Below the threshold of the localized field, although the enhanced THz field *E_THz_* shows a distribution with a maximum at the center region, the resonance mode (*f_res_*) and topology in terms of the current path are still identical to those of the simple slot without nanotips, owing to the high resistivity of the Si substrate (over 20000 Ωcm). Meanwhile, the nanotip structure can efficiently squeeze the mode volume inside the gap between the nanotips again, which can cause strong non‐equilibrium carrier transport (*J’*) under specific conditions above the threshold field. The nanotip structure, characterized by its unique geometry with tapered ends and sharp corners, offers suitable conditions for the confinement and manipulation of electromagnetic waves. As a result, the instantaneous potential energy applied to the vicinity of the nanotip structure in Si can result in tremendous current localization *J’*, effectively shutting off the fundamental resonance and creating a higher resonance spectrum, reaching the resonance mode of 2*f_res_*.

Because of the low photon energy at the level of a few meV, electrons in the valence band cannot acquire sufficient energy to overcome the bandgap of 1.14 eV through optical absorption. By colliding with an accelerated free electron via an excessively localized THz field, a bound electron under the valence band can obtain energy, as shown in Figure 1(b).^[^ ^37^ ^]^ To quantitatively describe this situation, the local conductivity of the Si around the nanotip structure was calculated as a function of the electric field of the THz wave, as shown in Figure 1(c) (Section S2, Supporting Information).^[^ ^21^, ^38^, ^39^ ^]^ The incident field strength corresponds to THz waves generated by an easily handled and widely used photoconductive antenna emitter driven by a femtosecond oscillator. Given that the incident THz field strength is only a few tens to hundreds of V/cm (Section S3, Supporting Information), the Si substrate can be assumed to be an insulator. By enhancing the field strength, the conductivity of the Si substrate increases exponentially. This dramatic increase in conductivity can only be expected well beyond the 40 kV cm^−1^ THz wave, which can be achieved by nanostructures with fields enhanced by over thousands of times relative to the incident field. This phenomenon is attributed to carrier multiplication, which results in a significant increase in carrier density and, consequently, a conductivity change, as shown in Figure 1(d). This process is similar to impact ionization, which creates new electron‐hole pairs. These successive sequential electron collisions transfer energy and further promote carrier multiplication. This implies that carrier multiplication can be generated by sufficient energy transfer from the significantly enhanced THz field in our nanotip samples beyond the threshold, even without phonon‐assisted interband transitions between different valleys.

**Figure 2:**
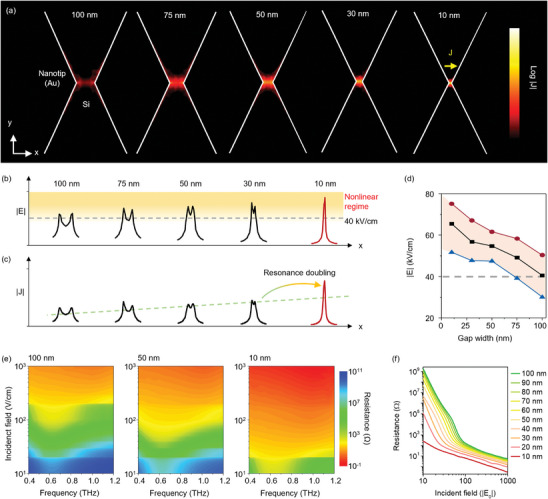
a) The calculated induced current across the nanotip gap was plotted in terms of the nanotip gap width. Dependence of b) enhanced electric field and c) induced current on the various gap widths. d) The absolute field amplitude plotted according to gap width. e) The correlations between the incident field and resistance of the nanotip gap for 100 nm, 50 nm, and 10 nm gap widths. f) The calculated resistance in terms of the incident field for various gap widths.

The redistribution of the THz field‐induced current density |*J*| in Si, which is affected by the presence of the nanotip structure, was investigated by comparing the gap widths of the nanotips (**Figure** **2**a). In this calculation, the incident THz field was assumed to 50 V cm^−1^. The THz field becomes strongly localized at the corners as the gap size decreases (100 nm to 10 nm). The localization indicates a significant field enhancement, implying carrier multiplication. The greatly enhanced THz field facilitates the acceleration of conduction band electrons, leading to the collision‐induced creation of new electron‐hole pairs. The threshold electric field that induced nonlinearity was determined to be 40 kV cm^−1^, as shown in the cross‐sectional field view in Figure 2(b). At a nanotip gap width of 100 nm, the field is mostly bounded at both corners of the end of each nanotip. As the gap width decreases (100 nm to 10 nm), the increased field is merged into a singular point; here, the gradational background illustrates the nonlinear regime. Accordingly, the induced current across the gap increases and reaches a maximum at a gap width of 10 nm (Figure 2c). The critical maximum, minimum, and average values of the induced field amplitude between the nanotips can be described in terms of the gap width (Figure 2d). In addition to the maximum strength, the minimum field affects the current channel between the nanotips, leading to a change in the resonance mode. For nanotip gaps smaller than 75 nm, the minimum strength of the localized field was greater than the threshold of 40 kV cm^−1^.

To obtain a deeper understanding of the parameters that play critical roles in inducing the charge current and resonance mode change, an analytical calculation of the resistance across the nanotip gap was performed (Section S4, Supporting Information). The resistance was considered only near the nanotip, where the strongly confined field around the gap effectively influences the conductivity of the substrate. This dramatic change in resistance can be explained in terms of the gap width of the THz spectra (Figure 2e). As the gap width decreases, the resistance around the resonance frequency approaches the lower threshold field amplitude, which appears as a correlation between the gap width and the resistance of the substrate. The change in resistance at the resonance frequency leading to the mode change is illustrated in Figure 2(f).

### 2.2 THz Resonance Mode Switching through Boosted Nonlinear Carrier Transport

**Figure 3:**
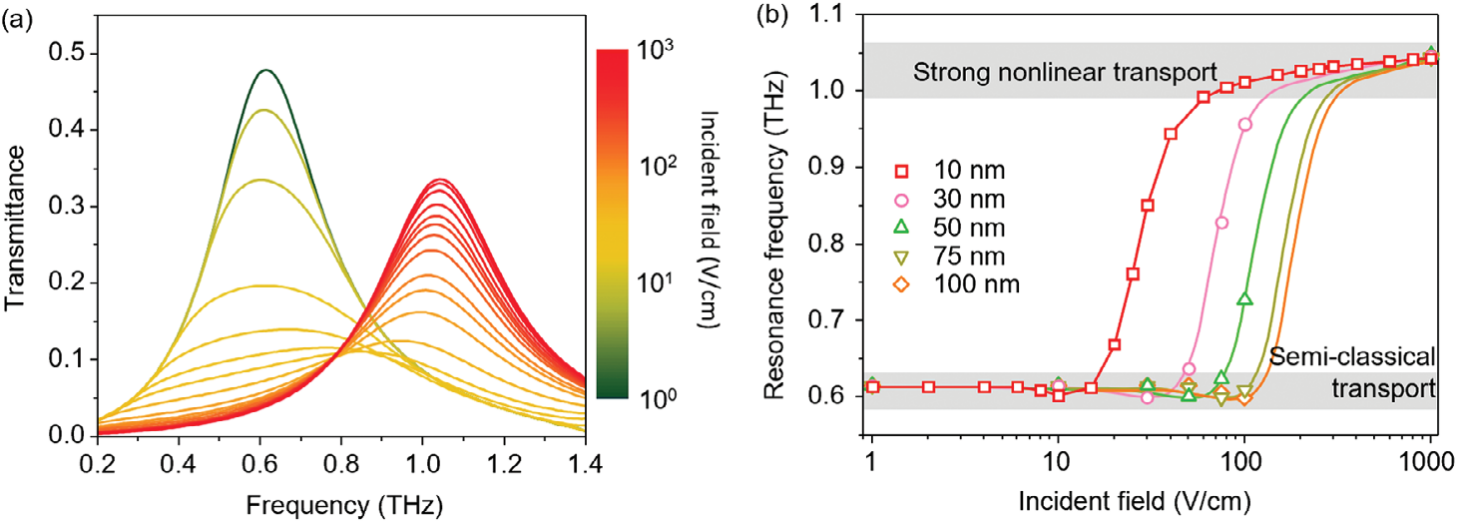
a) Transmittance spectra for the THz resonators and b) The resonance switching by the incident field for gap widths of 10, 30, 50, 75, and 100 nm.

To clarify the effect of field‐dependent microscopic carrier transport between the nanotips on macroscopic phenomena, we calculated the THz transmission spectra of the slot with nanotips using the finite element method, as shown in **Figure** **3**(a) (Section S1, Supporting Information). In this simulation, the gap size between the nanotips was supposed to 10 nm to achieve a clear resonance change under an unexaggerated incident field magnitude compared to that of a common THz system with a photoconductive antenna emitter. For the intrinsic initial carrier density in the substrate without a nonlinear response, the transmittance clearly indicated resonance for the metallic slot only. By increasing the incident field, the fundamental resonance corresponding to the slot was gradually suppressed and sequentially blue‐shifted. This spectral change due to the field‐dependent resistance change at the tiny spot on the slot center is similar to the resonance behavior of the pinch harmonics on a simple nanorod antenna (green‐to‐red transition in the spectrum).^[^ ^40^ ^]^ Under a field strength sufficient to decrease the resistance below a few tens of ohms inside the nanotip gap, as shown in Figure 2(e,f), the resonance frequency clearly doubled with the increase in transmittance, owing to the newly developed pathway for carriers. Note that abrupt transmittance changes and rapid resonance frequency increases occurred in the ranges of the incident THz field strength. Thus, with a sufficient field strength selection, we can utilize this phenomenon to develop advanced THz devices not only for broadband THz systems, such as THz time‐domain spectroscopy (THz‐TDS), but also for single‐frequency systems.

To help control this phenomenon of resonance transition depending on the incident field strength, we calculated the transmission spectra of metamaterials with various nanotip gaps. As the gaps became narrower, leading to a low field enhancement, the resonance transition required a more intense incident field strength, as shown in Figure 3(b). Abrupt transitions occurred under the weak THz waves allowed by a common photoconductive antenna, even with a 100 nm nanotip gap. The changes in transmittance also followed this trend.

**Figure 4:**
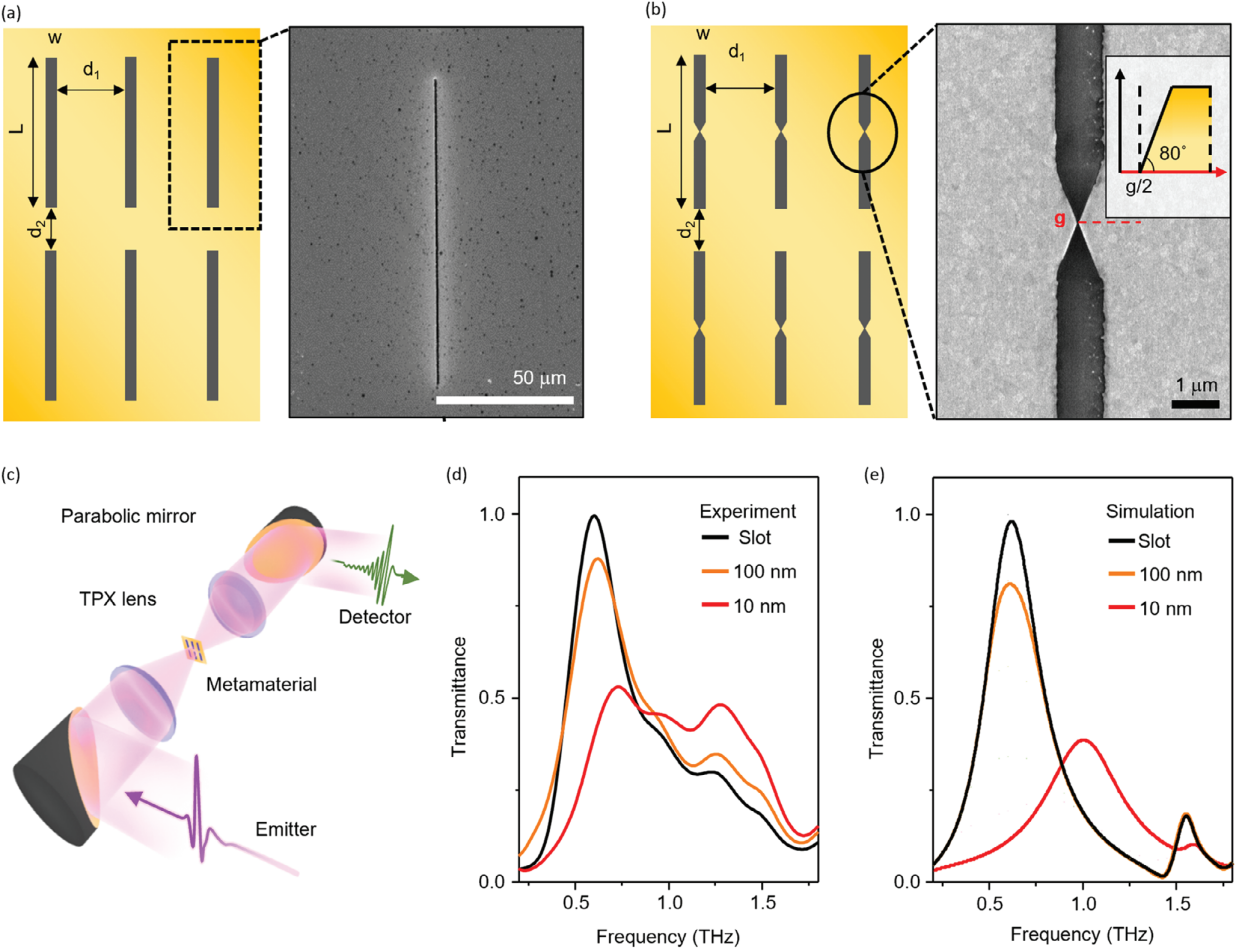
a) Schematics of fabricated nanoslot structures and enlarged SEM images for single unit slot. b) Schematics of fabricated nanoslot array with nanotip structure at the middle of the slot and SEM image. The inset provides a cross‐sectional image of the 3D nanotip structure. c) Experimental setup for transmission measurement. d) Measured transmittance spectra for bare slot, 10 nm nanotip, and 100 nm nanotip samples. e) Calculated spectra for the several gap width‐nanotip structures.

To experimentally obtain this dramatic resonance frequency modulation, we fabricated slot arrays with and without nanotip structures at their centers, as shown in **Figure** **4**(a,b). The patterns were made on a Cr/Au (5/95 nm) film, deposited on high‐resistivity silicon substrate (*ρ* > 20000 Ω), using photolithography for slots, and Ga^+^‐based focused ion beam (FIB) milling for nanotip structures (Section S5, Supporting Information). Identical slots with a length of 100 µm (*L*) and width of 1 µm (*w*) for 0.6 THz resonance were arranged in a 2×4 slot array with separations of 10 µm and 100 µm in the longitudinal and transverse directions, respectively, to avoid both Rayleigh minima and inter‐lattice resonance coupling.

We measured the transmission spectra of the slot arrays with and without nanotip structures in the THz range (0.2‐2.0 THz) using the THz‐time domain spectroscopy system, as shown in Figure 4(c). The THz electromagnetic waves emitted from the photoconductive antenna were focused onto the slot samples using a TPX lens pair. The focused THz wave was 1.2 mm in diameter, fully covering the slot samples. The transmitted THz waves were detected via electro‐optic sampling using a ZnTe crystal. The detected THz signal in the time domain was converted into a frequency‐domain spectrum via the fast Fourier transform. The transmitted field amplitude was obtained from the measured spectrum for the sample *E_sam_* divided by the spectrum for the bare Si substrate *E_sub_*; then, the square of the field amplitude gives the THz transmittance intensity as *T* = |*E_sam_*/*E_sub_* |^2^.

The measured THz transmittances for the slot only, the slot with a 100 nm nanotip gap, and the slot with a 10 nm nanotip gap are shown in Figure 4(d). The slot antenna without any fine structure shows a clear resonance at 0.6 THz (black). The fundamental resonance is determined by the relation $\lambda_{res}=\;2L\sqrt{1+{n_{s}}^{2}}$, where *λ_res_* is the resonance wavelength of 500 µm for 0.6 THz and *n_s_* is the refractive index of the Si substrate in the THz range. The slight decrease in transmittance can be attributed to the estimated effective THz field determined by the nanopatterns. This greatly enhanced field is thought to be responsible for the generation of nonlinear carrier transport, thereby affecting the overall transmittance of the system by disturbing the resonance behavior. A significant decrease in transmittance, accompanied by a noticeable blue shift in the resonance frequency, was observed for the 10 nm nanotip sample (shown in red). This drastic change in the THz spectra was responsible for the subsequent suppression of the resonance mode. The specific mechanism underlying the observed changes in the transmittance requires further investigation and analysis. To support the experimental observation of the suppression of the resonance mode, the transmission spectra of the slots with nanotips were calculated at an incidence of 75 V cm^−1^ (Figure 4e). The calculated spectra matched well with the experimental results, supporting the resonance change caused by the THz field‐dependent impact ionization phenomenon. Compared to the calculation in Figure 4(e), experimental results in Figure 4(d) show a broad spectrum that resembles multi‐resonance, particularly in the nanotip with a 10 nm gap. This could be attributed to the broadening caused by Gaussian beam‐shaped THz waves of our experimental system and slight fabrication variations in the nanotip gap within the slot array, which may have led to the nonlinear enhancement of the THz field being influenced by highly sensitive resonance changes under extreme conditions.

**Figure 5:**
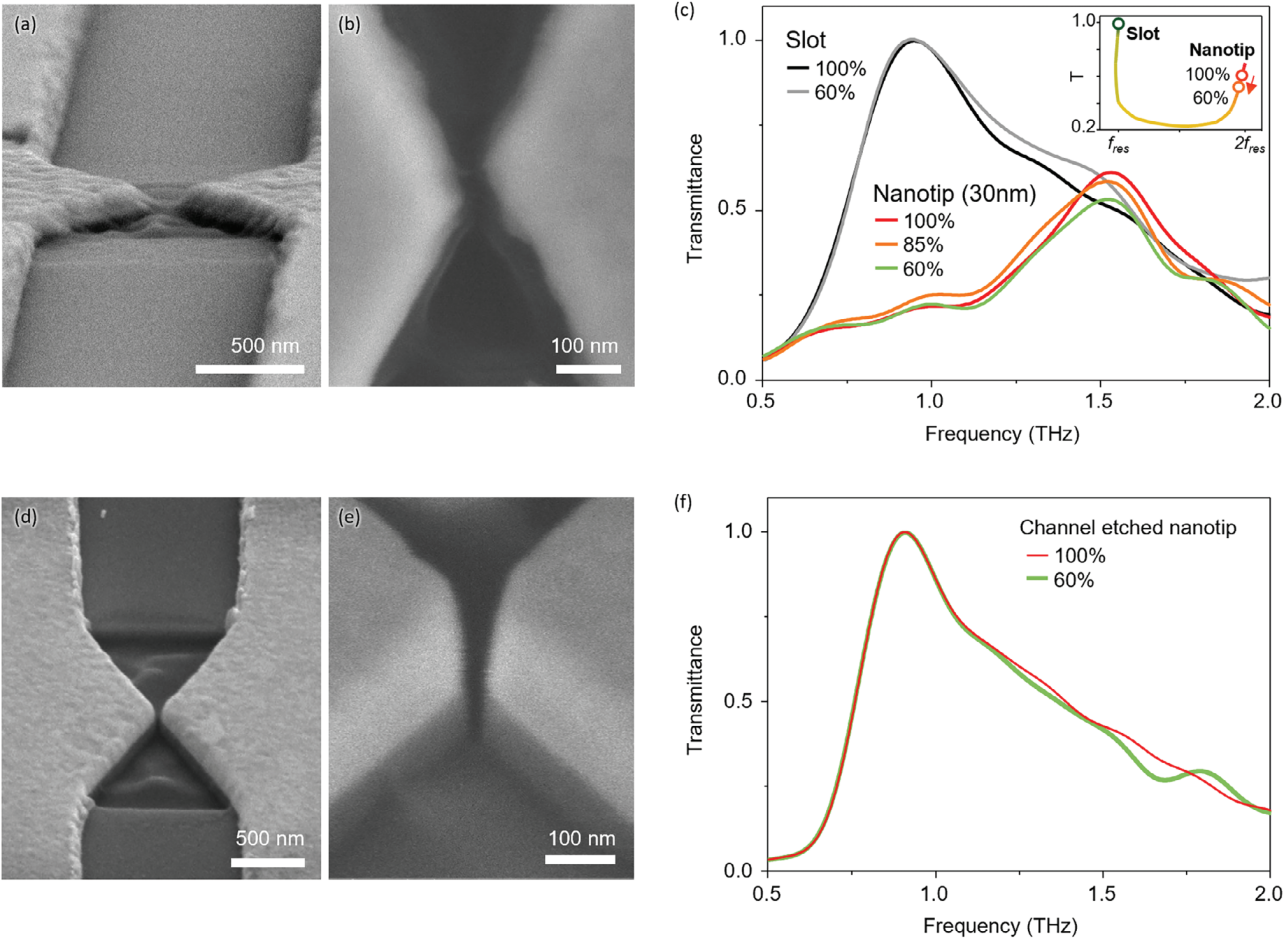
a,b) SEM image for the 30 nm gap. c) Measured THz transmittances for 1 µm width slot only and 1 µm slots with a 30 nm width nanotip under different field strengths. d,e) SEM image for the 30 nm gap with channel‐etched nanotips. f) THz transmittance of channel‐etched nanotips.

To understand the mechanism of the observed nonlinear behavior, the incident field‐dependent transmission spectra of the simple slot and the slot with nanotips were measured, as shown in **Figure** **5**. The 8 (2×4) slot arrays with or without nanotips were designed to have a fundamental resonance at 0.95 THz (*L* = 60 µm, *w* = 1 µm). At the center of the slot for nonlinear carrier transport, nanotips with 30 nm gaps were effectively fabricated, as shown in Figure 5(a,b). The simple slot exhibited field‐independent resonance, as shown in Figure 5(c). THz field amplitude is controlled down to 60% with consideration signal‐to‐noise ratio (Figure S6, Supporting Information). In contrast, the slot with the nanotip showed a decrease in resonance with a slight red shift upon decreasing the field. The resonance behaviors at the transmission maximum are summarized in the inset of Figure 5(c), with a tendency well‐matched to the resonance trace in Figure 3(b). To clarify the carrier transport phenomena between the nanotip edges, the gaps, which are the major paths of the current induced by the intense field enhancement on the metamaterial, were additionally etched using FIB, as shown in Figure 5(d,e). Although the nanotip still enhances the THz field by maintaining the metallic structure, the slot with the channel‐etched nanotip showed the fundamental resonance with THz field independency, as shown in Figure 5(f).

## 3 Discussion

**Figure 6:**
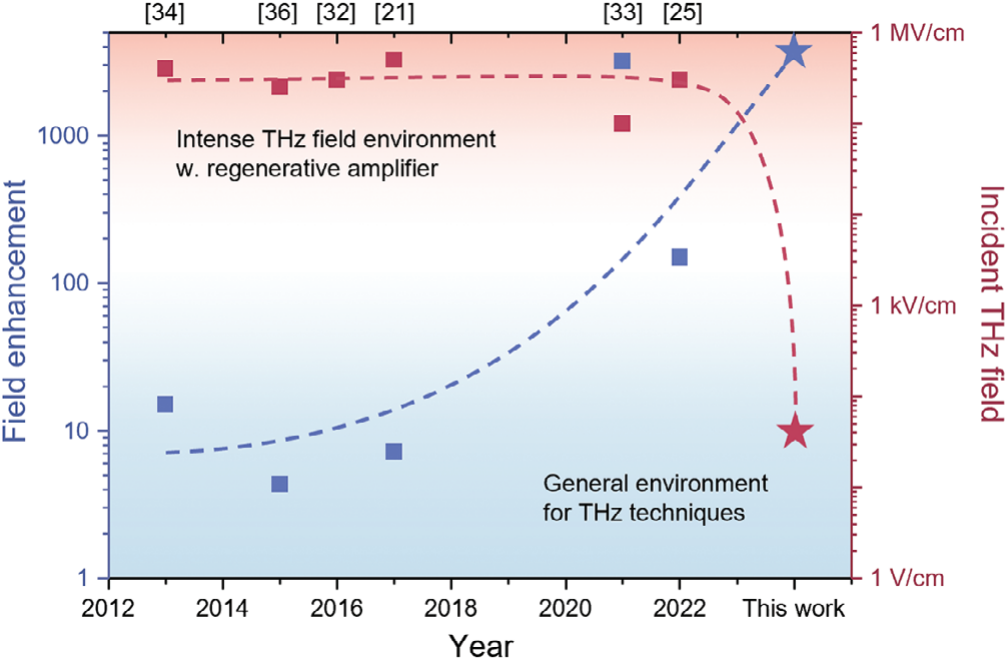
Overview of recent progress in research related to the resonance switching of metamaterials by nonlinear carrier dynamics on the substrate, in terms of field enhancement and required THz field strength.

We investigated nonlinear carrier transport in silicon induced by THz waves, which have photon energies hundreds of times smaller than the bandgap, using dynamic resonance mode switching on a nanostructured metamaterial. Unlike a simple nanoslot with field enhancement on the order of a few tens and static transmission spectra, the slot with nanotips showed changeable resonance behavior in terms of transmission amplitude and resonance frequency. This resonance behavior was induced by a decrease in the resistance to a few ohms, as shown in Figure 2(e,f), although the initial intrinsic carrier density was extremely suppressed in the substrate with very pure float‐zone silicon. To compare the recent progress, the trends in research related to resonance changes induced by field enhancement in metamaterials are summarized in **Figure** **6** (detailed in Table S1, Supporting Information). Although the magnitude of field enhancement has gradually increased, high‐power terahertz systems with electric field strengths exceeding several hundred kV/cm were required to induce these phenomena even in recent studies. In our study, we were the first to demonstrate nonlinear carrier transport phenomena using a metamaterial structure with an antenna featuring nanotips, which exhibits high electric field localization and a notable resonance shift, even under the relatively low and typical electric field strengths of femtosecond oscillator‐based THz‐TDS systems. By adopting sufficient metamaterials to allow electrode structures, the nanotip can be exploited to develop devices for the direct detection of THz waves without thermoelectricity or pyroelectricity.

Meanwhile, if an extremely strong field exceeding tens of MV/cm can be induced, carrier transport beyond the semi‐classical regime in the semiconductor substrate, as well as the direct current channel between nanotips caused by field emission, could contribute to resonance changes.^[^ ^41^, ^42^, ^43^ ^]^ To utilize this phenomenon, it is important to carefully consider the unimpeded electron transport in metallic tips within free space, along with the potential modification of the nanotip structure due to sustained field emission, such as through electromigration.

## 4 Conclusion

In conclusion, the suppression of the fundamental resonance mode by strong THz field confinement was observed using a 3D nanotip structure at the nanoscale under exceptionally low‐intensity THz electromagnetic waves. This remarkable accomplishment was achieved through the precise fabrication of a three‐dimensional nanoscale bowtie tip structure based on current state‐of‐the‐art nanotechnology. This induces the ultimate localization of the THz field at a point‐like small volume; consequently, the resistance of the substrate decreases to a few ohms, resulting in abnormal carrier multiplication, even for high‐resistivity Si. Our experimental discoveries were confirmed through a concretely designed simulation of the field map and induced current, along with the analytically calculated resistance of the substrate. These findings will provide profound implications and insights that promise to revolutionize advanced THz technologies beyond the quantum regime.

## Conflict of Interest

The authors declare no conflict of interest.

## Author Contributions

S.‐H.L. and M.K. contributed equally to this work. S.‐H.L. conceived the study, performed the experiments with Y.R., and conducted the optical simulation. M.K. fabricated the devices. M.‐K.K. and M.S. wrote the manuscript with S.‐H.L. and supervised the entire study. All authors contributed to the scientific discussion and manuscript revision.

## Supporting information



Supporting Information

## Data Availability

The data that support the findings of this study are available in the supplementary material of this article.

## References

[1] S.‐H. Lee , D. Lee , M. H. Choi , J.‐H. Son , M. Seo , Anal. Chem. 2019, 91, 6844. 31035757 10.1021/acs.analchem.9b01066

[2] K. A. Niessen , M. Xu , D. K. George , M. C. Chen , A. R. Ferré‐D'Amaré , E. H. Snell , V. Cody , J. Pace , M. Schmidt , A. G. Markelz , Nat. Commun. 2019, 10, 1026. 30833555 10.1038/s41467-019-08926-3 PMC6399446

[3] M. Sotome , M. Nakamura , T. Morimoto , Y. Zhang , G.‐Y. Guo , M. Kawasaki , N. Nagaosa , Y. Tokura , N. Ogawa , Phys. Rev. B 2021, 103, L241111.

[4] D. Zhao , H. Hu , R. Haselsberger , R. A. Marcus , M. E. Michel‐Beyerle , Y. M. Lam , J. X. Zhu , C. La‐O‐Vorakiat , M. C. Beard , E. E. M. Chia , ACS Nano 2019, 13, 8826. 31348643 10.1021/acsnano.9b02049

[5] D. Zhao , J. M. Skelton , H. Hu , C. La‐O‐Vorakiat , J. X. Zhu , R. A. Marcus , M. E. Michel‐Beyerle , Y. M. Lam , A. Walsh , E. E. M. Chia , Appl. Phys. Lett. 2017, 111, 201903.

[6] L. Luo , M. Mootz , J. H. Kang , C. Huang , K. Eom , J. W. Lee , C. Vaswani , Y. G. Collantes , E. E. Hellstrom , I. E. Perakis , C. B. Eom , J. Wang , Nat. Phys. 2023, 19, 201.

[7] J. E. Lee , J. Choi , T. S. Jung , J. H. Kim , Y. J. Choi , K. I. Sim , Y. Jo , J. H. Kim , Nat. Commun. 2023, 14, 2737. 37173319 10.1038/s41467-023-38422-8 PMC10182076

[8] Y. Kim , D. Kim , S.‐H. Lee , M. Seo , H.‐J. Jung , B. Kang , S.‐M. Lee , H.‐J. Lee , Opt. Express 2020, 28, 17143. 32679927 10.1364/OE.387783

[9] T. Otsuji , IEEE Trans. Terahertz Sci. Technol. 2015, 5, 1110.

[10] J. Tong , W. Zhou , Y. Qu , Z. Xu , Z. Huang , D. H. Zhang , Nat. Commun. 2017, 8, 1660. 29162817 10.1038/s41467-017-01828-2 PMC5698436

[11] L. Viti , D. Coquillat , D. Ercolani , L. Sorba , W. Knap , M. S. Vitiello , Opt. Express 2014, 22, 8996. 24787788 10.1364/OE.22.008996

[12] D. A. Bandurin , D. Svintsov , I. Gayduchenko , S. G. Xu , A. Principi , M. Moskotin , I. Tretyakov , D. Yagodkin , S. Zhukov , T. Taniguchi , K. Watanabe , I. V. Grigorieva , M. Polini , G. N. Goltsman , A. K. Geim , G. Fedorov , Nat. Commun. 2018, 9, 5392. 30568184 10.1038/s41467-018-07848-w PMC6300605

[13] C. Liu , L. Wang , X. Chen , J. Zhou , W. Hu , X. Wang , J. Li , Z. Huang , W. Zhou , W. Tang , G. Xu , S.‐W. Wang , W. Lu , Carbon NY 2018, 130, 233.

[14] S. Castilla , B. Terrés , M. Autore , L. Viti , J. Li , A. Y. Nikitin , I. Vangelidis , K. Watanabe , T. Taniguchi , E. Lidorikis , M. S. Vitiello , R. Hillenbrand , K. J. Tielrooij , F. H. L. Koppens , Nano Lett. 2019, 19, 2765. 30882226 10.1021/acs.nanolett.8b04171

[15] L. Viti , J. Hu , D. Coquillat , W. Knap , A. Tredicucci , A. Politano , M. S. Vitiello , Adv. Mater. 2015, 27, 5567. 26270791 10.1002/adma.201502052

[16] K. Peng , D. Jevtics , F. Zhang , S. Sterzl , D. A. Damry , M. U. Rothmann , B. Guilhabert , M. J. Strain , H. H. Tan , L. M. Herz , L. Fu , M. D. Dawson , A. Hurtado , C. Jagadish , M. B. Johnston , Science 2020, 368, 510. 32355027 10.1126/science.abb0924

[17] M. van Exter , D. Grischkowsky , Phys. Rev. B 1990, 41, 12140. 10.1103/physrevb.41.12140 9993669

[18] D. Grischkowsky , S. Keiding , M. van Exter , C. Fattinger , J. Opt. Soc. Am. B 1990, 7, 2006.

[19] P. Gaal , K. Reimann , M. Woerner , T. Elsaesser , R. Hey , K. H. Ploog , Phys. Rev. Lett. 2006, 96, 187402. 16712394 10.1103/PhysRevLett.96.187402

[20] F. H. Su , F. Blanchard , G. Sharma , L. Razzari , A. Ayesheshim , T. L. Cocker , L. V. Titova , T. Ozaki , J.‐C. J.‐C. Kieffer , R. Morandotti , M. Reid , F. A. Hegmann , Opt. Express 2009, 17, 9620. 19506611 10.1364/oe.17.009620

[21] A. T. Tarekegne , H. Hirori , K. Tanaka , K. Iwaszczuk , P. U. Jepsen , New J. Phys. 2017, 19, 123018.

[22] H. Hirori , A. Doi , F. Blanchard , K. Tanaka , Appl. Phys. Lett. 2011, 98, 091106.

[23] W. Kuehn , P. Gaal , K. Reimann , M. Woerner , T. Elsaesser , R. Hey , Phys. Rev. B 2010, 82, 075204. 10.1103/PhysRevLett.104.146602 20481951

[24] J. Hebling , M. C. Hoffmann , H. Y. Hwang , K.‐L. L. Yeh , K. A. Nelson , Phys. Rev. B Condens. Matter Mater. Phys. 2010, 81, 035201.

[25] B. J. Kang , D. Rohrbach , F. D. J. Brunner , S. Bagiante , H. Sigg , T. Feurer , Nano Lett. 2022, 22, 2016. 35133848 10.1021/acs.nanolett.1c04776

[26] S.‐H. Lee , D.‐K. Lee , C. Kim , Y. M. Jhon , J.‐H. Son , M. Seo , Opt. Express 2017, 25, 11436. 28788824 10.1364/OE.25.011436

[27] M. A. Seo , H. R. Park , S. M. Koo , D. J. Park , J. H. Kang , O. K. Suwal , S. S. Choi , P. C. M. Planken , G. S. Park , N. K. Park , Q. H. Park , D. S. Kim , Nat. Photonics 2009, 3, 152.

[28] M. A. Seo , A. J. L. Adam , J. H. Kang , J. W. Lee , S. C. Jeoung , Q. H. Park , P. C. M. Planken , D. S. Kim , Opt. Express 2007, 15, 11781. 19547541 10.1364/oe.15.011781

[29] N. Kim , S. In , D. Lee , J. Rhie , J. Jeong , D.‐S. Kim , N. Park , ACS Photonics 2018, 5, 278.

[30] H. Ryu , J. Kang , S. Lee , Results Phys. 2023, 54, 107049.

[31] M. Seo , J.‐H. Kang , H.‐S. Kim , J. Hyong Cho , J. Choi , Y. Min Jhon , S. Lee , J. Hun Kim , T. Lee , Q.‐H. Park , C. Kim , Sci. Rep. 2015, 5, 10280. 25998840 10.1038/srep10280 PMC4441136

[32] H. R. Seren , J. Zhang , G. R. Keiser , S. J. Maddox , X. Zhao , K. Fan , S. R. Bank , X. Zhang , R. D. Averitt , Light Sci. Appl. 2016, 5, e16078. 30167165 10.1038/lsa.2016.78 PMC6059934

[33] T. Dong , S. Li , M. Manjappa , P. Yang , J. Zhou , D. Kong , B. Quan , X. Chen , C. Ouyang , F. Dai , J. Han , C. Ouyang , X. Zhang , J. Li , Y. Li , J. Miao , Y. Li , L. Wang , R. Singh , W. Zhang , X. Wu , Adv. Funct. Mater. 2021, 31, 2100463.

[34] K. Fan , H. Y. Hwang , M. Liu , A. C. Strikwerda , A. Sternbach , J. Zhang , X. Zhao , X. Zhang , K. A. Nelson , R. D. Averitt , Phys. Rev. Lett. 2013, 110, 217404. 23745933 10.1103/PhysRevLett.110.217404

[35] J. Lee , J. Lee , G. Lee , D. Kim , Y. Ryu , M. Seo , Adv. Mater. 2024, 36, 2308975. 10.1002/adma.202308975 37994274

[36] A. T. Tarekegne , K. Iwaszczuk , M. Zalkovskij , A. C. Strikwerda , P. U. Jepsen , New J. Phys. 2015, 17, 043002.

[37] J. Bude , K. Hess , G. J. Iafrate , Phys. Rev. B 1992, 45, 10958. 10.1103/physrevb.45.10958 10001017

[38] A. G. Chynoweth , Phys. Rev. 1958, 109, 1537.

[39] W. Maes , K. De Meyer , R. Van Overstraeten , Solid State Electron 1990, 33, 705.

[40] H.‐R. Park , Y.‐M. Bahk , J. H. Choe , S. Han , S. S. Choi , K. J. Ahn , N. Park , Q.‐H. Park , D.‐S. Kim , Opt. Express 2011, 19, 24775. 22109504 10.1364/OE.19.024775

[41] G. Herink , L. Wimmer , C. Ropers , New J. Phys. 2014, 16, 123005.

[42] J. Zhang , X. Zhao , K. Fan , X. Wang , G. F. Zhang , K. Geng , X. Zhang , R. D. Averitt , Appl. Phys. Lett. 2015, 107, 231101.

[43] D. Matte , N. Chamanara , L. Gingras , L. P. R. De Cotret , T. L. Britt , B. J. Siwick , D. G. Cooke , Phys. Rev. Res. 2021, 3, 13137.

