# Supplementary material for: Ultra‐Low Threshold Resonance Switching by Terahertz Field Enhancement‐Induced Nanobridge
Source: Adv Sci (Weinh). 2024 Nov 4;12(1):2405225. doi: 10.1002/advs.202405225 (PMC11714150; doi:10.1002/advs.202405225)
Supplement: Supplementary file 1 — Supporting Information [file ADVS-12-2405225-s001.docx]

Supplementary information

**Ultra-low threshold resonance switching by terahertz field enhancement-induced nanobridge**

Sang-Hun Lee^1,†^, Moohyuk Kim^2,†^, Yeeun Roh^3^, Myung-Ki Kim^2,*^, and Minah Seo^2,3,*^

**Section S1: Distributions of THz field and induced current**

The nanoslots with nanotips were simulated with a structure as shown in Fig. S1. The permittivity of metal for the nano-slot and nanotips were described by the Drude model with the damping parameter and plasma frequency, 4.98 THz and 2.95 PHz, respectively, which give an optimized transmission spectrum to experimental results. The permittivity of the silicon was described as a lossy medium with conductivity in Fig. 1(c).

The distributions of current density and electric field depending on the incidence terahertz (THz) field were summarized in Fig. S2. For this simulation, the nanotips with 50 nm separation were placed at the center of the nanoslot, which has a 1 μm width and a length of 100 μm for resonance at 0.6 THz. By increasing the localized field magnitude, the induced current density becomes a circulation form via the nanotips located at the center of the nanoslot.


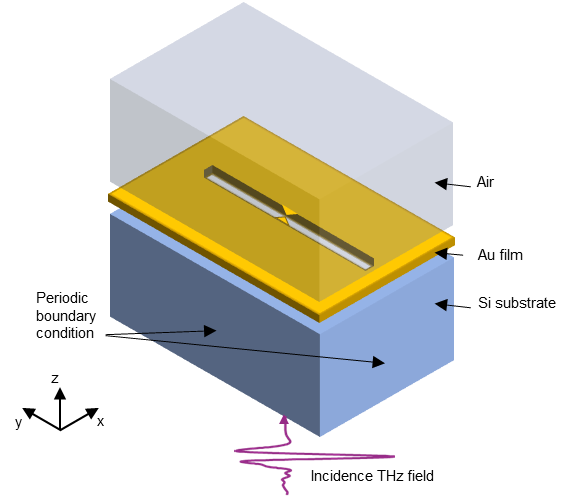


**Figure S1** Simulation schematic of the nanoslot


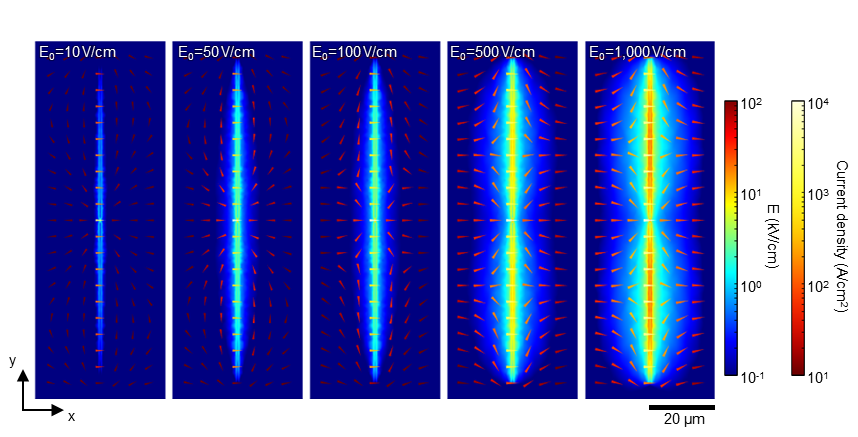


**Figure S2** Distributions of electric field and current density

**Section S2: THz Field-dependent conductivity of Si**

The impact ionization coefficient, *α*(*E*) = *α*_∞_*e^-b/E^*,^[S1]^ of high resistivity Si was from Ref ^[^*^S^*^2]^. From the definition of impact ionization coefficient, *α*=(*v*_n_ *n*)^-1^d*n*/d*t*, the carrier density in terms of THz electric field can be calculated as ln(*n*) = *α*(*E*)*v*_n_*t*_d_ + ln(*n*_0_), where *v*_n_ *­*is the drift velocity (*v*_n_=10^7^cm/s), *t*_d_ is the THz pulse duration (*t*_d_ =1ps), *n*_0_ is initial electron density (*n*_0_ = 2.2×10^11^_­_ cm^-3­­­^ for Si resistivity of 20 kΩcm). Thus, the conductivity can be derived by *σ*=*enμ*, where *μ* is the electron mobility of Si (*μ*=1400 cm^2^/Vs).

**Section S3: Quantification of THz Field amplitude in THz-TDS**

The amplitude of the incidenct THz electric field, which was measured by the electro-optic sampling with ZnTe crystal, was calculated using Eq. S1: ^[S3, S4]^

$\frac{I_{x}-I_{y}}{I_{x}+I_{y}}=\frac{2\pi n_{0}^{3}r_{41}t_{THz}t_{optics}d}{\lambda_{0}}E_{THz}$ Eq. S1

, where *I_x_* and *I_y_* are the signal levels of photodetectors, *n*_0_ is the refractive index of ZnTe at 800 nm (*n*_0_=2.85), *d* is thickness of ZnTe (*d*=2mm), *r*_41_ is the electro-optic coefficient of the ZnTe (*r*_41_=3.9 pm/V), *t*_THz_ is THz transmission coefficient for ZnTe (*t*_THz_ = 0.48), *λ*­_0_ is the wavelength of the laser (*λ*­_0_=800 nm) *(S5)*. *t*_optics_ is a simplified transmission coefficient of THz optics from a sample to ZnTe crystal, which includes transmission by two TPX lens and two ITO mirrors. The amplitude of the THz pulse is shown in Fig. S3. The THz wave is generated using the photoconductive antenna, which has a 50 μm gap fabricated on a low-temperature grown GaAs, driven with optical pumping of 100 mW and an electric bias of 100V_p-p_. The THz wave showed a peak-to-peak amplitude of 37.7 V/cm.

**Figure S3** Electric field amplitude of the THz pulse

**Section S4: Resistance between nanotips**

The nanotips were fabricated on the gold film with an electric connection, direct measurement and calculation of resistance across the nanotips are not suitable to investigate the carrier multiplication effect. Thus, the resistance was estimated inside the localized area changing conductivity by THz field enhancement. The resistance inside an arbitrary small spherical volume, *V’,* centered on the gap between nanotips is calculated. A radius of the calculated volume, *V’,* is 5 μm radius, which fully covered the conducting region affected by the field enhancement in Si substrate. By a simple conduction model between a resistor of cross-sectional area *A* and length *x*, the resistance between the nanotips can be lead as Eq. S2:

$\frac{1}{R_{gap}}=\frac{1}{x^{2}}\int_{V'} \sigma(|E|)dV$ Eq. S2

, where *x* is the gap width between nanotips. In Fig. S4, resistivity-increased Si areas near the nanotips under the incidence THz field, *E_0,_* were depicted. The semi-insulating Si region without conductivity change are marked in white. As the incident field strength increases, the region where the resistivity decreases expands, contributing to the formation of induced current channels between the nanotips, resulting in resonance switching, as shown in Fig. S5.


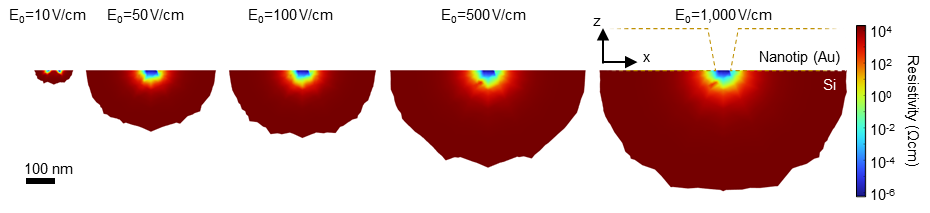


**Figure S4** Resistivity change of Si substrate by THz field.

**
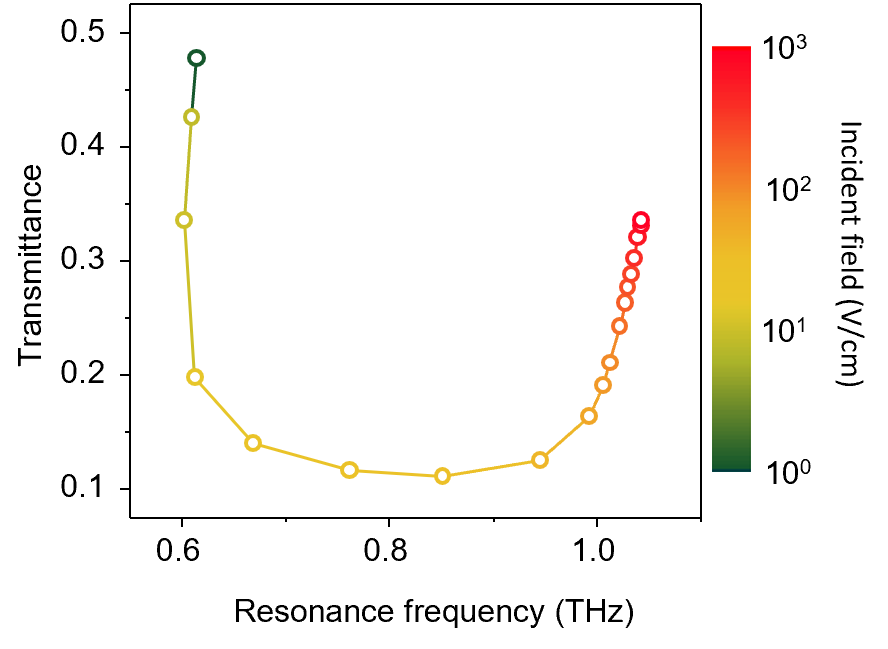
**

**Figure S5** Resonance change by incident fields

**Section S5: Fabrication of metamaterials with nanotips**

The metamaterials with nanotips were fabricated on a chromium/gold (Cr/Au) film, with a 5/95 nm thickness, layered atop a high-resistivity silicon wafer (with resistivity greater than 20,000Ω), utilizing an electron beam evaporator system for deposition. The typical metamaterial slot configurations, measuring 100 µm in length and 1 µm in width, were developed through a maskless photolithography process coupled with lift-off techniques. These metamaterials were systematically organized into a 2×4 grid, maintaining 40 µm and 72 µm spacings on the horizontal and vertical axes, respectively.

A specialized variant of these structures, equipped with nanotips, followed the same fabrication process as the standard 100/1 µm slot metamaterials. The distinctive feature in this process was the central segment of the photolithography pattern—1 µm wide and 2 µm length—left unpatterned to facilitate the following focused ion beam (FIB) milling procedure, as depicted in Fig. S6. To construct a nanotip, we applied a proximal milling method using a Gallium ion (Ga^+^) based FIB system, specifically the ThermoFisher Scientific Helios G4 UC model. This technique capitalized on the inherent Gaussian proximity effect during FIB milling. Accordingly, we fabricated patterns intentionally smaller than the envisaged nanotip dimensions to accommodate the ion beam’s proximity effect, which can influence areas within several tens of nanometers when etching a gold film of 100 nm in depth. As shown in Fig. S6(c), we fabricated the structures by adjusting the spacing *s* between two triangular patterns to 0 nm, 5 nm, and 30 nm, respectively. Figure S6(d) demonstrates how the nanotip's gap size varies in accordance with *s*. By increasing *s*, the smallest gap size between nanotips along *x*-direction can be decreased. Further, we fine-tuned the etching depth to meticulously preserve the silicon channels adjacent to the nanotips.


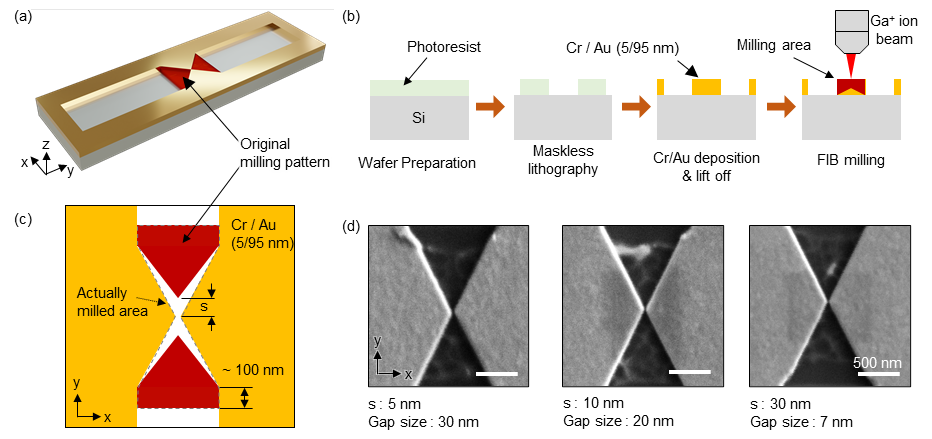


**Figure S6** Fabrication of metamaterials with nanotips. (a,c) Schematic of Proximal focused-ion-beam (FIB) milling techniques, where the red region represents the milling area. (b) Overview of the entire fabrication process for metamaterials with 3D nanotips (d) SEM images of fabricated 3D nanotips with tiny gaps.

**Section S6: Experimental setup for THz-TDS**

We measured THz field strength dependence of the THz metamaterials with nanotips using THz-TDS experimental setup with optical power control for the PCA emitter, as shown in Fig. S7. Optical power was decreased using a pair of linear polarizers; one was with fixed angle matched to the polarization direction of the fs laser source, another was with sufficient rotation angle. Thus, optical power is decreased by following the Malus’ law, as shown in Fig. S8(a). Actual THz amplitude in the time-domain by incident optical power into the PCA emitter was well controlled with direct linear correlation as shown in Fig. S8(b). To verify power independency in THz spectra of PCA emitter, correlation coefficients with emitted THz signal by reduced optical pump and THz signal with maximum optical pump to PCA emitter. By comparing the correlation coefficient, we can compare both spectral and phase change briefly. All signals showed good correlation with a coefficient reaching 1, which means all signals only changed amplitude without any spectral and phase changes. Therefore, we could measure the field strength dependency of the THz metamaterial with nanotips with THz waves in Fig. S8(d).


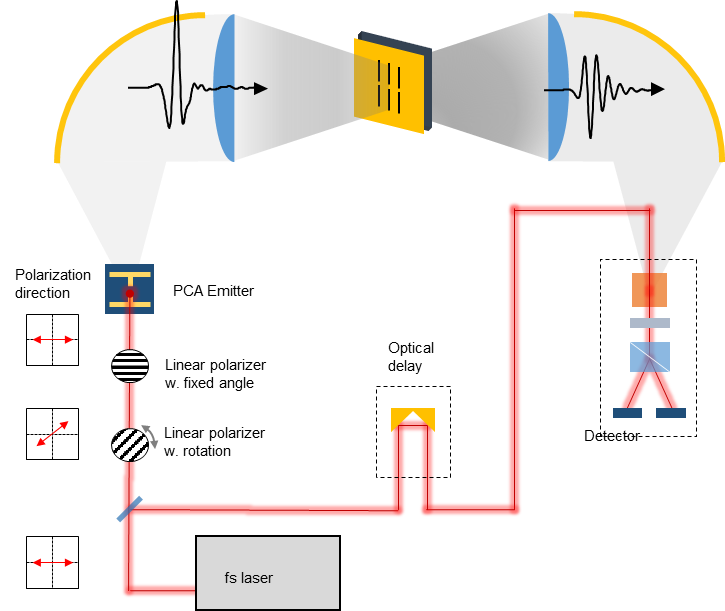


**Figure S7** Scheme of the experimental setup for THz-TDS.

**
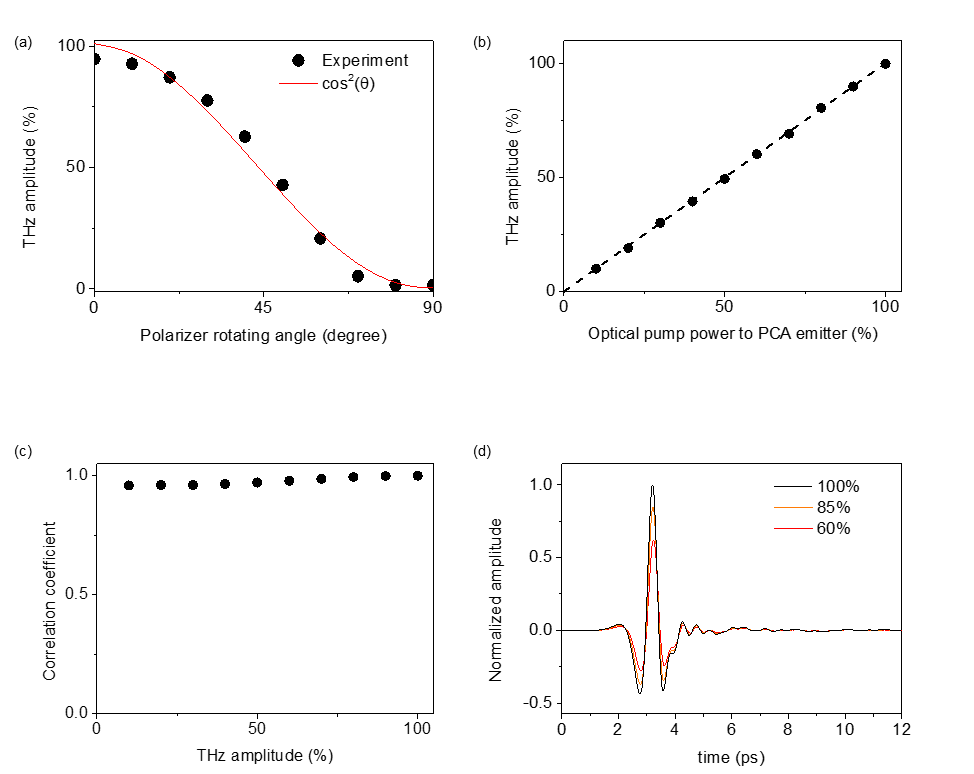
**

**Figure S8** (a) THz amplitude by rotation angle of the polarizer. (b) THz amplitude by optical pump power into PCA emitter. (c) correlation coefficient with amplitude-controlled THz wave and the THz signal with maximum amplitude. (d) THz waveforms for measurement of field strength dependence on THz metamaterial with nanotips.

**Section S7: Recent progress of the metamaterial resonance switching by nonlinear carrier transport**

| **Year** | **Ref.** | **Type** | **Substrate** | **\|E_0_\|**  **(kV/cm)** | **Field enhancement** | **Resonance shift** |
| --- | --- | --- | --- | --- | --- | --- |
| 2013 | [34] | SRR | GaAs | 400 | 15 | ~ 0.03 THz |
| 2015 | [36] | Rod antenna | Si | 250 | 4.32 | - |
| 2016 | [32] | InAs disk | GaAs | 300 | - | < 0.01 GHz |
| 2017 | [21] | Rod antenna | Si | 500 | 7.2 | - |
| 2021 | [33] | SRR | Si | 100 | 3200 | 0.17 THz |
| 2022 | [25] | SRR | Ge, GaAs, Si, ZnS | 300 | 150 | ~ 0.1 THz |
| 2024 | This work | Nanotips on  slot antenna | Si | 37.7×10^-3^ | 3750,  1312 (with IMI) | ~ 0.5 THz |

Table S1. Summary of recent research related to the resonance switching of metamaterials by nonlinear carrier dynamics on the substrate.

**Reference**

[S1] A. G. Chynoweth, Physical Review 1958, 109, 1537.

[S2] A. T. Tarekegne, H. Hirori, K. Tanaka, K. Iwaszczuk, P. U. Jepsen, New J Phys 2017, 19, 123018.

[S3] H. Hirori, A. Doi, F. Blanchard, K. Tanaka, Appl Phys Lett 2011, 98, 091106.

[S4] Q. Wu, X. C. Zhang, Appl Phys Lett 1997, 71, 1285.

[S5] P. C. M. Planken, H.-K. Nienhuys, H. J. Bakker, T. Wenckebach, Journal of the Optical Society of America B 2001, 18, 313.
